# Prognostic value of ‘late’ electroencephalography recordings in patients with cardiopulmonal resuscitation after cardiac arrest

**DOI:** 10.1007/s00415-021-10549-y

**Published:** 2021-04-19

**Authors:** Jakob I. Doerrfuss, Alexander B. Kowski, Martin Holtkamp, Moritz Thinius, Christoph Leithner, Christian Storm

**Affiliations:** 1 Department of Neurology and Experimental Neurology, Charité—Universitätsmedizin Berlin, Berlin, Germany; 2 Department of Nephrology and Medical Intensive Care, Charité—Universitätsmedizin Berlin, Berlin, Germany

**Keywords:** EEG, Predictive value of tests, Resuscitation, Outcome, Targeted temperature management

## Abstract

**Background:**

Electroencephalography (EEG) significantly contributes to the neuroprognostication after resuscitation from cardiac arrest. Recent studies suggest that the prognostic value of EEG is highest for continuous recording within the first days after cardiac arrest. Early continuous EEG, however, is not available in all hospitals. In this observational study, we sought to evaluate the predictive value of a ‘late’ EEG recording 5–14 days after cardiac arrest without sedatives.

**Methods:**

We retrospectively analyzed EEG data in consecutive adult patients treated at the medical intensive care units (ICU) of the Charité—Universitätsmedizin Berlin. Outcome was assessed as cerebral performance category (CPC) at discharge from ICU, with an unfavorable outcome being defined as CPC 4 and 5.

**Results:**

In 187 patients, a ‘late’ EEG recording was performed. Of these patients, 127 were without continuous administration of sedative agents for at least 24 h before the EEG recording. In this patient group, a continuously suppressed background activity < 10 µV predicted an unfavorable outcome with a sensitivity of 31% (95% confidence interval (CI) 20–45) and a specificity of 99% (95% CI 91–100). In patients with suppressed background activity and generalized periodic discharges, sensitivity was 15% (95% CI 7–27) and specificity was 100% (95% CI 94–100). GPDs on unsuppressed background activity were associated with a sensitivity of 42% (95% CI 29–46) and a specificity of 92% (95% CI 82–97).

**Conclusions:**

A ‘late’ EEG performed 5 to 14 days after resuscitation from cardiac arrest can aide in prognosticating functional outcome. A suppressed EEG background activity in this time period indicates poor outcome.

**Supplementary Information:**

The online version contains supplementary material available at 10.1007/s00415-021-10549-y.

## Introduction

Predicting neurological outcome after cardiopulmonal resuscitation (CPR) following cardiac arrest (CA) is important for determining further treatment choices and decisions regarding withdrawal of life-sustaining therapy. Diagnostic workup after CA to assess prognosis consists of clinical examination, short-latency somatosensory evoked potentials (SSEPs), measurement of neuron-specific enolase (NSE), brain imaging and electroencephalography (EEG). This outcome prediction is especially important in patients who remain comatose after resuscitation from CA [1].

Several EEG parameters are associated with a poor prognosis after CA [2]. Recent studies have suggested that ‘early’ continuous EEG can detect poor outcome with higher sensitivity than ‘late’ EEG [3, 4]. In early EEG measurements, the sensitivity for the prediction of unfavorable outcome decreased after > 12 h following resuscitation from CA while the specificity remained robust [4]. However, EEG measurements are not broadly available at all times. EEG activity underlies a natural evolution following CA and could also be altered due to effects of sedative medication [5, 6]. This has become of particular importance since the routine implementation of targeted temperature management (TTM). According to current guidelines, almost all survivors after CA not responding to painful stimuli should receive TTM in a range of 32–36 °C for at least 24 h, which requires administration of sedatives [1].

Current guidelines provide different recommendations regarding the timing of EEG recordings for the assessment of prognosis after CA: an advisory statement from the European Resuscitation Council from 2015 recommends measuring EEG 72 h after recovery of spontaneous circulation (ROSC) [7]. In contrast, the task force for Belgian recommendations suggests measuring EEG ‘as soon as possible’ [8].

In this study, we seek to determine the clinical significance of a ‘late’ EEG recording between days 5 and 14 after resuscitation from CA in comatose patients. We hypothesize that the evaluation of such a ‘late’ EEG can aide in predicting functional outcome and that the predictive value of ‘late’ EEG recordings is higher in patients not under continuous sedative medication.

## Methods

### Data source and patients

We analyzed data from consecutive adult patients (aged 18 or above) admitted to the medical intensive care units (ICU) of the Charité—Universitätsmedizin Berlin (at Campus Virchow-Klinikum) after successful resuscitation from CA between June 1st 2009 and May 31st 2013. At that time, EEG recordings performed between days 5 and 14 after successful resuscitation from CA were part of clinical routine. Late EEG recordings were requested by ICU physicians only in patients still comatose. Institutional approval for this retrospective, observational study was provided by the local Ethics committee (EA2/115/13).

Clinical data were derived from a large prospective database described previously [9]. These data comprised age at CA, sex, location of CPR (out-of-hospital vs. in-hospital), initial cardiac rhythm (shockable vs. non-shockable), time to ROSC (tROSC), NSE at day 3 after CA, SSEP data (24 h to 4 days post CA), EEG data (5 to 14 days post CA), and CPC score at discharge. CPC scores were determined at the time of ICU discharge and prospectively documented in the database. Electroencephalography and SSEP data were assessed following strict criteria by three of the authors (JD, AK, MT) and, in uncertain cases, reassessed by two other authors (MH, CL).

### Primary and secondary outcomes

Our primary study outcome parameter was cerebral performance category (CPC) at discharge from the ICU. We dichotomized CPC, defining a CPC score of 1–3 as favorable outcome and a CPC of 4 and 5 as unfavorable neurological outcome. This was done to avoid falsely allocating patients with prolonged recovery to the unfavorable outcome group. To improve comparability to other studies, we performed a supplementary analysis where a CPC score of 3 (severe cerebral disability) at discharge was considered an unfavorable outcome.

### Definition of parameters

#### EEG

**Fig. 1:**
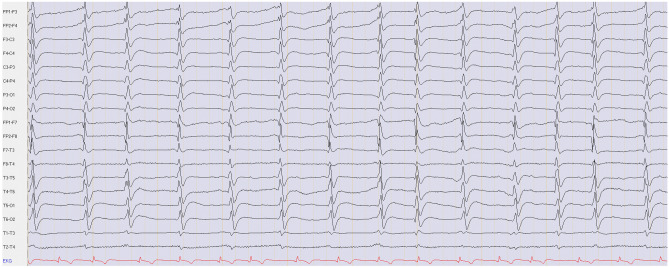
Example of GPDs on suppressed background activity

5 to 14 days after CA, a ‘late’ EEG with video-monitoring was performed for 20 min using a digital 21-channel recording system (Nihon Kohden, Japan) with the international 10–20 system for electrode placement and reviewed with standard montages. EEG data were analyzed for four predefined features that were previously described to be associated with unfavorable outcome after CPR [10]:

1. *Suppressed background activity without discharges* was defined as amplitude < 10 µV, during the entire recording, resulting in non-assessable frequency, without disruption by focal or generalized discharges.
2. *Generalized periodic discharges (GPDs) on suppressed background activity* was defined as amplitude of background activity < 10 µV during the entire recording which was interrupted by GPDs defined according to the American Clinical Neurophysiology Society’s (ACNS) Standardized Critical Care EEG Terminology. GPDs are characterized by periodically recurring monomorphic discharges with waveforms of a duration < 0.5 s regardless of number of phases or waveforms lasting ≥ 0.5 s with a maximum of three phases [11]. An example of GPDs on suppressed background activity is given in Fig. 1.
3. *GPDs on unsuppressed background activity* was defined as amplitude of background activity ≥ 10 µV which was interrupted by GPDs.
4. *Burst-suppression pattern* (BSP) was defined in accordance to the ACNS standardized critical care EEG terminology as generalized periodic, recurring high-voltage discharges occurring with more than three phases and a duration of ≥ 0.5 s against a suppressed background activity [11].

##### SSEPs

*Median nerve somatosensory evoked potentials* (SSEPs) were recorded using Nihon Kohden Neuropack four mini system (Nihon Kohden, Japan). Stimulation intensity was adjusted to produce a peripheral response (thumb twitch). For each recording > 500 SSEPs were performed and averaged. The highest amplitude of a reproducible cortical potential (> 4.5 ms after the N13 peak) was measured (peak to peak or baseline to peak). N20 was only determined as bilaterally absent, if cortical noise level was below 0.25 µV and there were no reproducible cortical potentials but reproducible spinal and peripheral potentials. SSEPs were recorded 24 h to 4 days after resuscitation from CA.

#### NSE

Serum concentration of NSE was determined at day 3 after CA. A threshold of 90 µg/l was applied for calculation of sensitivities and specificities [12].

### Standardized care at the intensive care units

All patients were treated with TTM following a strict in-house protocol along the lines of the International Liaison Committee on Resuscitation recommendations [13]. The target temperature of 33 °C was maintained for 24 h followed by a controlled re-warming rate of 0.25 °C per hour. Fever had to be avoided for further 72 h. In all patients, a computer controlled surface cooling device (Arctic Sun™ Temperature Management System, C.R.BARD, Colorado, USA) was used. A combination of intravenous midazolam and fentanyl or isoflurane (volatile) and remifentanil was used for analgosedation.

### Withdrawal of life-sustaining therapy

Withdrawal of life-sustaining therapy was always based on careful consideration of multimodal neurological prognostication and a considerable observation period. In 2011, an internal protocol for prognostication was established [14] and modified according to new evidence over time. The protocol is largely in line with the 2014 guidelines of the European Society of Intensive Care Medicine [7] and emphasizes multimodal prognostication and an observation period of at least 7 days in most cases. Patients’ advanced directives and preferences communicated by relatives were taken into account. In a relevant subset of patients, withdrawal of life-sustaining therapy was not performed despite poor prognostic findings. These patients were discharged in coma or unresponsive wakefulness syndrome. We followed these patients in long-term and found no case of an unexpected late recovery, indicating a low probability of a self-fulfilling prophecy for our prognostication algorithm [15].

### Statistical analysis

Categorical variables were analyzed with Pearson’s *χ*^2^ or Fischer’s test. Continuous data were checked for normal distribution using Kolmogorov–Smirnov test and were then presented as mean ± standard deviation or median and interquartile range (IQR) where appropriate; Mann–Whitney *U* test was used for continuous variables.

Sensitivity and specificity were calculated with 2 × 2 contingency tables and are presented with 95% confidence intervals (CI). For the identification of independent predictors for unfavorable outcome at ICU discharge, a binary logistic regression analysis (inclusion method: stepwise backward, *p* < 0.1 [*p* in], *p* < 0.05 [*p* out], iteration 20, cutoff set 0.26 and constant was included) was used with clinically relevant covariates to estimate odds ratios (OR) with 95% CI. Parameters with quasi-complete separation were excluded from the logistic regression analysis.

Statistical analyses were performed with SPSS version 25 (IBM, Chicago, IL, USA).

## Results

### Patient population

**Fig. 2:**
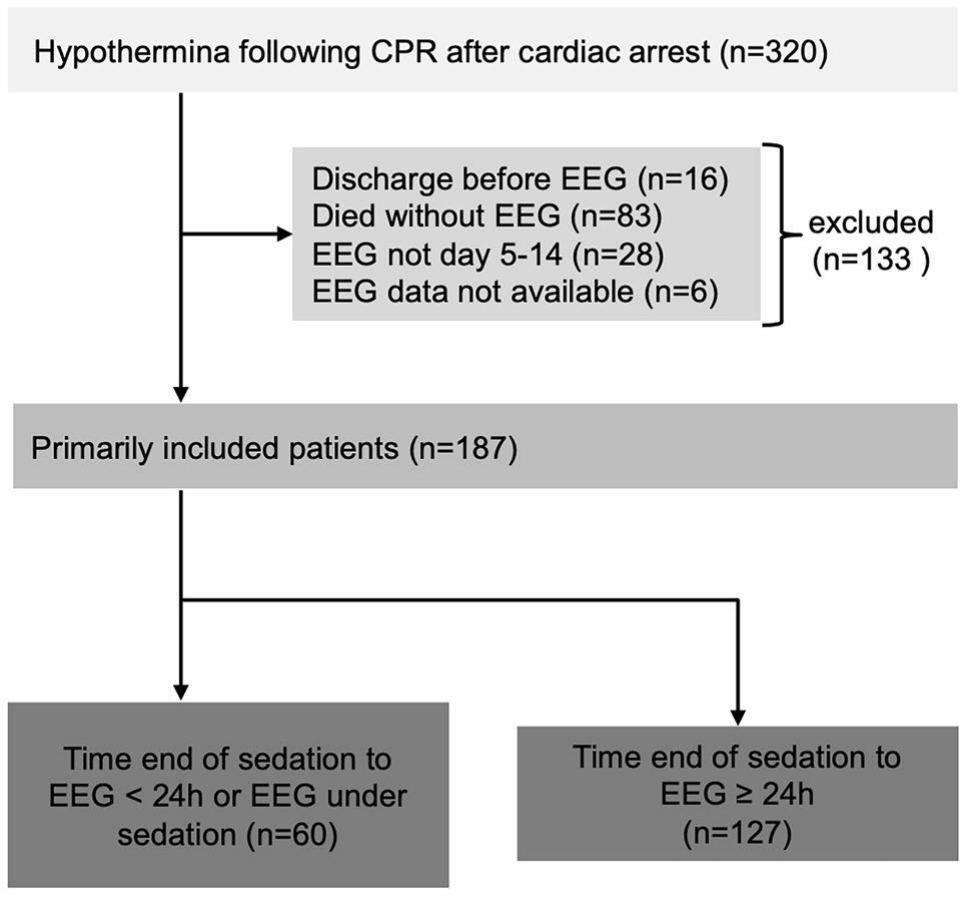
Patient recruitment

During the 4-year study period, 320 patients were treated with TTM after out-of-hospital or in-hospital CA and successful resuscitation. One hundred and thirty-three patients were excluded from the analysis because they were discharged or had died before the EEG was performed, because no EEG was recorded in the time window from day 5–14 after CA or because EEG recordings could not be retrieved (Fig. 2).

Our final analysis included 187 patients, mean age was 62 ± 16 years, 28% female, 71% with out-of-hospital CPR. Median CPC at discharge was 2 (interquartile range (IQR) 1–5), 41% of patients had an unfavorable outcome at discharge, and mortality rate was 27%. Median time point of EEG recording was 8 days after CA (IQR 7–10), and median ICU length of stay was 21 days (IQR 13–33).

Comparing included and excluded patients, we found no significant differences concerning sex, age, and location of CPR. However, the initial presence of non-shockable rhythm, time to ROSC, mean NSE, CPC, and mortality showed significant group-differences (Table-e1, online only).

Of all 187 included patients, 127 were without continuous administration of sedative agents for at least 24 h before the EEG recording. In 60 patients, the EEG recording was performed under or within 24 h of continuous administration of sedatives. There were no statistically significant differences between both groups with regards to sex, age, proportion of out-of-hospital CPR and initial shockable rhythm, NSE at day 3, and clinical outcome (Table e-2, online only).

### Outcome prediction in patients without continuous administration of sedatives for > 24 h before EEG measurements

In the 127 patients without continuous administration of sedatives, 55 patients (43%) had an unfavorable outcome.

Seventeen patients with unfavorable outcome (31%) had a continuously suppressed background activity with no discharges. A suppressed background activity with no discharges predicted unfavorable outcome with a sensitivity of 31% (95% confidence interval [CI] 20–45) and a specificity of 99% (95% CI 91–100). GPDs on unsuppressed background activity were associated with a sensitivity of 42% (95% CI 29–56) and a specificity of 92% (95% CI 82–97) for the prediction of unfavorable outcome.

One patient with suppressed background activity without discharges and six patients with GPDs on unsuppressed background had a favorable outcome. Two of the patients with GPDs on unsuppressed background and favorable outcome had received a bolus of sedatives in the 24 h preceding the EEG recordings. The patient with suppressed background activity without discharges and favorable outcome was a 67 old female who was resuscitated from CA following pulmonal artery embolism. Sedation with midazolam and fentanyl was discontinued 4.5 days before the EEG recording and no bolus of sedative medication was given in the 48 h preceding the EEG recording. We provide a more detailed description of this case in the supplementary material.

No false positives were present in the eight patients who showed both GPDs and suppressed background activity (GPDs on suppressed background; sensitivity for the prediction of unfavorable outcome 15% (95% CI 7–27); specificity 100% (95% CI 94–100). The prognostic parameters are summarized in Table 1; the analyses for an unfavorable outcome being defined as CPC 3–5 are provided in the supplementary material (Table e3, online only).

**Table 1:** Predictive values for unfavorable outcome (CPC 4 and 5)

	Frequency (*n*) (*n* = 127)	Sensitivity (95% CI)	Specificity (95% CI)	True positive	False positive	True negative	False negative
*Patients without continuous sedative-administration for at least 24 h*							
EEG parameters							
Suppressed background without discharges	14% (18)	31% (20–45)	99% (91–100)	17	1	71	38
GPDs on suppressed background	6% (8)	15% (7–27)	100% (94–100)	8	0	72	47
GPDs on unsuppressed background	23% (29)	42% (29–56)	92% (82–97)	23	6	66	32
Other parameters							
NSE > 90 μg/l	19% (24)	42% (29–56)	98% (91–100)	23	1	71	32
Bilateral absent SSEPs	14% (18)	33% (21–47)	100% (94–100)	18	0	72	37
*Patients with continuous sedative-administration stopped less than 24 h before or with EEG during continuous sedation*							
EEG parameters							
Suppressed background without discharges	8% (5)	23% (9–46)	100% (89–100)	5	0	38	17
GPDs on suppressed background	5% (3)	14% (4–36)	100% (89–100)	3	0	38	19
GPDs on unsuppressed background	20% (12)	27% (12–50)	84% (68–93)	6	6	32	16
Other parameters							
NSE > 90 μg/l	15% (9)	32% (15–55)	95% (81–99)	7	2	36	15
Bilateral absent SSEPs	7% (4)	18% (6–41)	100% (89–100)	4	0	38	18

*CPC* cerebral performance category at intensive care unit discharge, *CI* confidence interval, *GPDs* generalized periodic discharges, *NSE* neuron-specific enolase, *SSEPs* short-latency somatosensory evoked potentials

The presence of suppressed background activity without discharges (OR 36.9, 95% CI 3.3–414.3) and of GPDs on unsuppressed background (OR 57.3, 95% CI 8.4–390.8) both independently predicted unfavorable outcome after CA, see Table 2 (for CPC 3–5 as unfavorable outcome see Table e4, online only).

**Table 2:** Predictors for CPC-based outcome (CPC 1–3 vs 4 and 5)

	CPC-based outcome		Univariate analyses	Binary logistic regression, Exp(B) [95% CI]
	Favorable (CPC 1–3)	Unfavorable (CPC 4 and 5)		
	*n* = 72	*n* = 55		
*Patients without continuous sedative-administration for at least 24 h*				
Demographics and basic information:				
Female sex, *n* (%)	17 (24)	19 (35)	*p* = 0.233^a^	Not included
Age [years], median (IQR)	63 (52–73)	70 (61–75)	*p* = 0.052^b^	Not significant
Out-of-hospital CPR, *n* (%)	47 (69)	37 (77)	*p* = 0.403	Not included
Initial rhythm: not shockable, *n* (%)	18 (32)	30 (67)	***p* = 0.001**^**a**^	**5.9** [1.3–26.5]
TROSC [min], median (IQR)	12 (7.5–20)	18 (10–28)	*p* = 0.055^b^	Not significant
NSE day 3				
[µg/l], median (IQR)	20 (15–27)	68 (33–130)	***p* < 0.001**^**b**^	Not included
NSE > 90 μg/l, *n* (%)	1 (1)	23 (42)	***p* < 0.001**^**a**^	**56.3** [4.5—704.7]
Electrophysiology:				
EEG				
Suppressed background without discharges, *n* (%)	1 (1)	17 (31)	***p* < 0.001**^**a**^	**36.9** [3.3 – 414.3]
GPDs on suppressed background, *n* (%)	0 (0)	8 (15)	***p* < 0.001**^**a**^	Not included^†^
GPDs on unsuppressed background, *n* (%)	6 (8)	23 (42)	***p* < 0.001 **^**a**^	**57.3** [8.4–390.8]
SSEP				
SSEPs bilateral absent, *n* (%)	0 (0)	18 (33)	***p* < 0.001**^**c**^	Not included^†^

	*n* = 38	*n* = 22		
*Patients with continuous sedative-administration stopped less than 24 h before or with EEG during continuous sedation*				
Demographics and basic information:				
Female sex, *n* (%)	11 (29)	6 (27)	*p* = 1.000^a^	Not included
Age [years], median (IQR)	61 (51–70)	61 (55–73)	*p* = 0.634^b^	Not significant
Out-of-hospital CPR, *n* (%)	25 (66)	15 (75)	*p* = 0.559^a^	Not included
Initial rhythm: not shockable, *n* (%)	10 (31)	9 (53)	*p* = 0.218^a^	Not significant
TROSC [min], median (IQR)	12 (10–20)	19 (12–24)	*p* = 0.118^b^	Not significant
NSE day 3				
[µg/l], median (IQR)	21 (13–28)	44 (26–111)	***p*** **<** **0.001**^b^	Not included
NSE > 90 μg/l, *n* (%)	2 (5)	7 (32)	***p*** **= 0.009**^a^	**6.6** [1.1–39.1]
Electrophysiology:				
EEG				
Suppressed background without discharges, *n* (%)	0 (0)	5 (23)	***p* = 0.005**^a^	Not included^†^
GPDs on suppressed background, *n* (%)	0 (0)	3 (14)	***p* = 0.045**^a^	Not included^†^
GPD on unsuppressed background, *n* (%)	6 (16)	6 (27)	*p* = 0.327^a^	Not significant
SSEP				
SSEPs bilateral absent, *n* (%)	0 (0)	4 (18)	***p*** **= 0.015**^a^	Not included^†^

The *p*-values and odds-ratios of statistically significant parameters are written in bold

*CPC *cerebral performance category at intensive care unit discharge, *IQR* interquartile range, *tROSC* time to resuscitate spontaneous circulation, *NSE* neuron-specific enolase, *CPR* cardiopulmonal resuscitation

^a^Fisher’s exact (two-sided)

^b^Mann–Whitney *U* test (two-sided)

^c^Pearson’s *χ*^2^ test (two-sided). *Exp(B)* odds ratio; *CI* confidence interval

^†^The variables were not included to avoid quasi-complete separation in the model

In the patient group without continuous administration of sedatives for at least 24 h before the EEG measurement, 24 patients (19%) had NSE values > 90 μg/l 3 days after resuscitation from CA. The sensitivity for the prediction of an unfavorable outcome in patients with NSE > 90 μg/l was 42% (95% CI 29–56) and specificity was 98% (95% CI 91–100). One patient with NSE > 90 μg/l had a favorable outcome at discharge (CPC 1).

In the same patient group, SSEPs were bilateral absent in 18 patients (14%). In no patient with favorable outcome, SSEPs were bilateral absent, resulting in a sensitivity for this outcome parameter of 33% (95% CI 21–47) and a specificity of 100% (95% CI 94–100).

Late EEG correctly predicted unfavorable outcome in 23 patients in whom SSEPs were not bilaterally absent and NSE was ≤ 90 μg/l (4 patients with GPDs on unsuppressed background, 7 patients with suppressed background without discharges and 12 patients with GPDs on unsuppressed background). SSEPs were able to correctly predict unfavorable outcome in one patient with normal EEG and NSE ≤ 90 μg/l. In all patients with unfavorable outcome and NSE > 90 μg/l, either SSEPs or EEG parameters were also predictive of unfavorable outcome. In five patients with CPC > 3, no prognostic parameter was predictive of this unfavorable outcome.

### Outcome prediction in patients with continuous sedative-administration stopped < 24 h before or with EEG during continuous sedation

Of the 60 patients with EEG recording within 24 h after administration of sedatives or with EEG recording during continuous sedation, 22 patients (37%) had an unfavorable outcome. In this patient group, the sensitivity for the prediction of unfavorable outcome was lower for the EEG parameters ‘suppressed background without discharges’ and ‘GPDs on unsuppressed background’ parameters as compared to the group of patients with EEG without continuous administration of sedatives. The diagnostic accuracy of the EEG parameter ‘GPDs on suppressed background’ was not different between the two groups. Specificities of all outcome predictors were comparable between both patient groups (see Table 1). The analysis of predictors for CPC-based outcome is shown in Table 2.

Only three patients had a burst-suppression-pattern at the time of EEG recording. Therefore, we omitted this EEG pattern from our statistical analysis.

### Outcome prediction with unfavorable outcome defined as CPC 3–5

When an unfavorable outcome was more broadly defined as CPC 3–5, five patients shifted to the unfavorable outcome group (four patients without continuous administration of sedatives for at least 24 h before the EEG measurement, one patient with sedative administration). With this alternative definition, 59 patients (46%) without continuous sedative-administration had an unfavorable outcome. In this patient group, suppressed background without discharges predicted unfavorable outcome with a sensitivity of 29% (95% CI 18–42) and a specificity of 99% (95% CI 91–100). GPDs on suppressed background had a sensitivity of 14% (95% CI 6–26) and a specificity of 100% (95% CI 93–100). The sensitivity for GPDs on unsuppressed background was 41% (95% CI 28–54) and the specificity was 93% (95% CI 83–97).

All results with this alternative outcome definition including predictive values for all parameters and analysis of predictors of CPC-based outcome are listed in the supplementary material (Tables e3 and e4).

## Discussion

In this study, a suppressed background activity without discharges in a ‘late’ EEG recording 5–14 days after CPR following CA had a specificity of 99% for the prediction of unfavorable outcome in patients without continuous administration of sedative medication for at least 24 h prior to the EEG recording. When both GPDs and a suppressed background activity (GPDs on suppressed background) were present, there were no false-positives, although the number of patients in this group was small (*n* = 9), resulting in a broader confidence interval. The predictive values of these EEG parameters were comparable to bilateral absent SSEPs 24 h to 4 days post CA or to a NSE > 90 µg/l 3 days after CA.

With a specificity of 92% and a false-positive rate of 8%, the predictive value of GPDs on unsuppressed background activity does not seem suitable as a parameter for the detection of an unfavorable outcome after CPR.

The sensitivities of the EEG parameters ‘suppressed background activity without discharges’ and ‘GPDs on unsuppressed background activity’ were lower in patients in whom continuous administration of sedatives was stopped less than 24 h before the EEG recording while GPDs on unsuppressed background did not seem to be affected by sedative medication. While the sensitivities of most outcome predictors were reduced in patients under influence of sedative medication, the specificity of these outcome predictors remained mostly similar.

EEG plays an important role in prognostication after CA, and its importance has not declined since the introduction of TTM into clinical routine [10, 16, 17]. The diagnostic yield of EEG recordings in patients with TTM is time-dependent [4–6]. Focusing on a ‘late’ EEG recording, presumably associated with a lower load of sedative drugs, our study extends prior reports on time specificity of EEG patterns to 5–14 days after CA.

One patient with suppressed EEG background activity without discharges, who was not sedated during or 24 h before the EEG measurement, had a favorable functional outcome at discharge. This result is surprising as in other studies no patient with EEG background activity < 10 µV had a favorable functional outcome [3, 18]. In these studies, EEG was performed within 24 h after cardiac arrest. While low voltage EEG < 20 µV is present in up to 10% of the general population and is often considered a normal variant, suppressed EEG background activity (< 10 µV) is regarded as a malignant EEG feature unequivocally associated with unfavorable outcome [19, 20]. We provide a more detailed description of the patient in the supplementary material. Summing up, the clinical course of the patient provides no clear explanation for the suppressed background activity. However, we cannot entirely exclude that the patient was administered a bolus of sedatives in the hours prior to EEG recording which was not documented in the electronic chart.

Suppressed background activity without discharges and the presence of GPDs on unsuppressed background both independently predicted unfavorable outcome. The presence of one of these parameters correctly predicted unfavorable outcome in 11 patients in whom SSEPs and NSE were unpredictive of unfavorable outcome. The pathophysiology underlying GPDs is not yet fully understood. GPDs are considered to be a consequence of loss of inhibitory interneurons, which are in particular vulnerable to hypoxia [21, 22]. The predictive value of GPDs in patients with hypoxic ischemic brain damage has been described previously in a group of 119 patients after CA in whom the EEG was performed within the first 14 days (mean 3.8 days) after the event [23]. In that study, a similar rate of GPDs after CA was detected.

In comparison to other studies in which an EEG was performed early after CA, there was a somewhat higher rate of suppressed background activity with GPDs [10, 17] and suppressed background activity without discharges [17] in our patients with unfavorable outcome. In contrast to studies with EEG examinations performed early after CA [10, 17], burst suppression patterns were only rarely detected in our study (*n* = 3). Our findings could hint towards an increased relevance of GPDs and a suppressed background activity in late EEG recordings, when these EEG patterns are not modified by sedative drugs which are required during TTM. This hypothesis corresponds with our finding of a diminished sensitivity for both EEG parameters in patients under sedative medication. These results are also in line with a recently published study focusing on EEG reactivity, the significance of this parameter for the prediction of unfavorable outcome after CA was reduced in patients under sedative medication [24]. The reduced sensitivity of EEG parameters in patients under sedative medication could also be explained alternatively: patients with reduced brain activity could be in less ‘need’ of sedative medication while patients with more brain activity could require more sedative medication. Thus, sedation would not be the cause of a reduced sensitivity for the detection of unfavorable outcome but it would be a sign of a certain amount of brain activity which might be associated with unfavorable outcome but might be high enough to prevent suppressed background activity.

BSPs, especially those with ‘identical bursts’, recorded in the first 72 h after CA are an important predictor for unfavorable outcome [6]. However, it was demonstrated that these BSPs evolve into less specific EEG patterns early after CA [25], which may explain their low frequency in our patient population.

There are limitations to consider. First, a comparison of the results of an ‘early’ vs. a ‘late’ EEG recording would have been desirable. Second, we only have data on outcome at ICU discharge and no data on long-term outcome. To avoid assigning patients with prolonged recovery falsely to the ‘unfavorable outcome group’, we considered a CPC of 3 to be a favorable outcome. There were no relevant changes to our results when the assignment of a CPC of 3 was changed to the unfavorable outcome group. Third, although we accounted for sedative drugs, we did not assess intake of classical antiseizure medication, which about 9% of ICU patients receive [26] and which could also have influenced EEG findings [27]. Fourth, we cannot exclude that in patients in whom sedatives had been withdrawn ≥ 24 h before the EEG recording, there was still some effect of these lipophilic substances after prolonged release and redistribution from fatty tissue. What is more, in 28 subjects (22%) in the patient group without continuous administration of sedatives, a bolus administration of either benzodiazepines or propofol was documented, which could also have influenced EEG results. Fifth, assessing outcome late after CA in a clinical study bears the risk of selection bias. At 5 days after CA, a considerable proportion of patients with severe hypoxic encephalopathy have already died. Likewise, it is possible that patients with mild hypoxic encephalopathy already have been discharged from the ICU at this time point. Therefore, we cannot rule out that our study includes a relatively low proportion of patients with either severe or very mild hypoxic encephalopathy. Thus, our patient selection most likely differs from those of studies on early EEG after CA. This could be an explanation for the relatively high proportion of patients with suppressed EEG background activity. Lastly, a major possible confounding factor in most studies assessing prognostication after CA is that of self-fulfilling prophecy as the results of the EEG examinations were known to the caregivers and, therefore, are likely to have influenced decisions regarding continuation or withdrawal of further therapy [28].

In summary, suppressed background activity without discharges and suppressed background activity with GPDs in a ‘late’ EEG recording 5–14 days after resuscitation from CA predict unfavorable outcome in patients not under continuous administration of sedatives. A relevant minority of patients with GPDs on unsuppressed background activity may regain consciousness within the next days.

## Supplementary Information

Below is the link to the electronic supplementary material.

Supplementary file1 Additional supplementary material: Detailed description of the patient with suppressed background activity and favorable outcome at discharge. (DOCX 35 kb)

## Data Availability

The data that support the findings of this study are available on request from the corresponding author. The data are not publicly available due to privacy or ethical restrictions.
