# Supplementary material for: Prognostic value of ‘late’ electroencephalography recordings in patients with cardiopulmonal resuscitation after cardiac arrest
Source: J Neurol. 2021 Apr 19;268(11):4248–57. doi: 10.1007/s00415-021-10549-y (PMC8505381; doi:10.1007/s00415-021-10549-y)
Supplement: Supplementary file 1 — Supplementary file1 Additional supplementary material: Detailed description of the patient with suppressed background activity and favorable outcome at discharge. (DOCX 35 kb) [file 415_2021_10549_MOESM1_ESM.docx]

**Table e1 - Clinical variables in included and excluded patients**

|  | **included subjects** (n=187) | **excluded subjects** (n=133) | **univariate analyses** |
| --- | --- | --- | --- |
| **Demographics and basic information:** |  |  |  |
| Female sex, n (%) | 53 (28) | 35 (26) | p=0.705^a^ |
| Age [years], median (IQR) | 64 (52-73) | 67 (56-75) | p=0.230 |
| Out-of-hospital CPR, n (%) | 124 (71) | 86 (70) | p=0.797^a^ |
| Initial rhythm: not shockable, n (%) | 67 (45) | 69 (68) | **p<0.001^a^** |
| TROSC [min], median (IQR) | 14 (9-24) | 18 (12-30) | **p=0.022^b^** |
| **NSE day 3 [µg/l],** median (IQR) | 26 (18-60) | 48 (21-176) | **p=0.001^b^** |
| **Outcome:** |  |  |  |
| **Cerebral performance category (CPC), n (%)** |  |  | **p<0.001^c^** |
| CPC 1 - returned to normal cerebral function | 70 (37) | 16 (12) |  |
| CPC 2 - disability but sufficient function for independent activity of daily life | 35 (19) | 7 (5) |  |
| CPC 3 - severe disability | 5 (3) | 0 (0) |  |
| CPC 4 - coma | 26 (14) | 3 (3) |  |
| CPC 5 - death | 51 (27) | 105 (80) |  |
| **Unfavorable outcome [CPC 4-5], n (%)** | 77 (41) | 108 (83) | **p<0.001^a^** |
| **Death [CPC 5], n (%)** | 51 (27) | 105 (80) | **p<0.001^a^** |

N – number; IQR – interquartile range; tROSC – time to resuscitate spontaneous circulation; NSE – neuron specific enolase; CPR – cardio-pulmonal-resuscitation. ^a^Fisher´s exact (2-sided); ^b^Mann-Whitney-U test (2-sided); ^c^Pearson’s Chi-squared test (2-sided).

**Table e2 - Clinical variables in patients without continuous sedative-administration for at least 24 h and patients with continuous sedative-administration stopped less than 24 h before or with EEG during continuous sedation**

|  | **no sedative administration**  (n=127) | **sedative administration**  (n=60) | **univariate analyses** |
| --- | --- | --- | --- |
| **Demographics and basic information:** |  |  |  |
| Female sex, n (%) | 36 (28) | 17 (28) | p=1.0^a^ |
| Age [years], median (IQR) | 67 (52-74) | 61 (52-71) | p=0.111^b^ |
| Out-of-hospital CPR, n (%) | 84 (72) | 40 (69) | p=0.723^a^ |
| Initial rhythm: not shockable, n (%) | 48 (48) | 19 (39) | p=0.382^a^ |
| TROSC [min], median (IQR) | 14 (8-24) | 15 (10-20) | p=0.728^b^ |
| **NSE day 3 [µg/l],** median (IQR) | 28 (19-61) | 24 (16-52) | p=0.231^b^ |
| **Outcome:** |  |  |  |
| **Cerebral performance category (CPC), n (%)** |  |  | p=0.757^c^ |
| CPC 1 - returned to normal cerebral function | 47 (37) | 23 (38) |  |
| CPC 2 - disability but sufficient function for independent activity of daily life | 21 (17) | 14 (23) |  |
| CPC 3 - severe disability | 4 (3) | 1 (2) |  |
| CPC 4 - coma | 18 (14) | 8 (13) |  |
| CPC 5 - death | 37 (29) | 14 (23) |  |
| **Unfavorable outcome [CPC 4-5], n (%)** | 55 (43) | 22 (37) | p=0.429 ^a^ |
| **Death [CPC 5], n (%)** | 37 (29) | 14 (23) | p=0.483^a^ |

N – number; IQR – interquartile range; tROSC – time to resuscitate spontaneous circulation; NSE – neuron specific enolase; CPR – cardio-pulmonal-resuscitation. ^a^Fisher´s exact (2-sided); ^b^Mann-Whitney-U test (2-sided); ^c^Pearson’s Chi-squared test (2-sided).

**Table e3 Sensitivities, specificities, and predictive values for the prediction of unfavorable functional outcome (CPC 3-5)**

|  | **Frequency (n)**  **(n=127)** | **Sensitivity (95%CI)** | **Specificity**  (95% CI) | **True positive** | **False Positive** | **True-negative** | **False-negative** |
| --- | --- | --- | --- | --- | --- | --- | --- |
|  | ***Patients without continuous sedative administration for at least 24 h*** | | | | |  |  |
| **EEG parameters** |  |  |  |  |  |  |  |
| Suppressed background without discharges | 14% (18) | 29% (18-42) | 99% (91-100) | 17 | 1 | 67 | 42 |
| GPDs on suppressed background | 6% (8) | 14% (6-26) | 100% (93-100) | 8 | 0 | 68 | 51 |
| GPDs on unsuppressed background | 23% (29) | 41% (28-54) | 93% (83-97) | 24 | 5 | 63 | 35 |
| **Other parameters** |  |  |  |  |  |  |  |
| NSE > 90 µg/l | 19% (24) | 39% (27-53) | 99% (91-100) | 23 | 1 | 67 | 36 |
| Bilateral absent SSEPs | 14% (18) | 31% (20-44) | 100% (93-100) | 18 | 0 | 68 | 41 |
|  |  |  |  |  |  |  |  |
| ***Patients with continuous sedative-administration stopped less than 24 h before or with EEG during continuous sedation*** | | | | | | | |
|  | **Frequency (n)**  **(n=60)** | **Sensitivity (95%CI)** | **Specificity**  (95% CI) | **True positive** | **False Positive** | **True-negative** | **False-negative** |
| **EEG parameters** |  |  |  |  |  |  |  |
| Suppressed background without discharges | 8% (5) | 22% (8-44) | 100% (88-100) | 5 | 0 | 37 | 18 |
| GPDs on suppressed background | 5% (3) | 13% (3-35) | 100% (88-100) | 3 | 0 | 37 | 20 |
| GPDs on unsuppressed background | 20% (12) | 26% (11-49) | 84% (67-93) | 6 | 6 | 31 | 17 |
| **Other parameters** |  |  |  |  |  |  |  |
| NSE > 90 µg/l | 15% (9) | 30% (14-53) | 95% (80-99) | 7 | 2 | 35 | 16 |
| Bilateral absent SSEPs | 7% (4) | 17% (6-40) | 100% (88-100) | 4 | 0 | 37 | 19 |

CPC – cerebral performance category at intensive care unit discharge; CI ­– confidence interval; GPDs - generalized periodic discharges; NSE – neuron specific enolase; SSEPs - short-latency somatosensory evoked potentials

**Table e4 – Predictors for CPC-based outcome (CPC 1-2 vs CPC 3-5)**

|  | **CPC based outcome** | | **univariate**  **analyses** | **binary logistic**  **regression^§^,**  **Exp(B)** [95% CI] |
| --- | --- | --- | --- | --- |
|  | **favorable**  **(CPC 1-2)** | **unfavorable**  **(CPC 3-5)** |  |  |
| ***Patients without continuous sedative administration for at least 24 h***  n=68 n=59 | | | | |
| **Demographics and basic information:** |  |  |  |  |
| Female sex, n (%) | 15 (22) | 21 (36) | p=0.115^a^ | not included |
| Age [years], median (IQR) | 63 (52-73) | 70 (61-75) | **p=0.031^b^** | not significant |
| Out-of-hospital CPR, n (%) | 45 (70) | 39 (75) | p=0.677 | not included |
| Initial rhythm: not shockable, n (%) | 16 (31) | 32 (65) | **p=0.001^a^** | 5.0 [1.3 - 19.3] |
| TROSC [min], median (IQR) | 12 (8-20) | 16 (10-27) | p=0.077^b^ | not included |
| **NSE day 3** |  |  |  |  |
| [µg/l], median (IQR) | 16 (15-27) | 62 (32-125) | **p<0.001^b^** | not included |
| NSE > 90 µg/l, n (%) | 1 (2) | 23 (39) | **p<0.001^a^** | **35.3** [3.1 - 404.1] |
| **Electrophysiology:** |  |  |  |  |
| **EEG** |  |  |  |  |
| Suppressed background without discharges, n (%) | 1 (2) | 17 (29) | **p<0.001^a^** | **26.1** [2.5 – 267.9] |
| GPDs on suppressed background, n (%) | 0 (0) | 8 (14) | **p<0.001^a^** | not included† |
| GPDs on unsuppressed background, n (%) | 5 (7) | 24 (41) | **p<0.001 ^a^** | **83.5** [8.7-795.1] |
| **SSEP** |  |  |  |  |
| SSEPs bilateral absent, n (%) | 0 (0) | 18 (31) | **p<0.001^c^** | not included^†^ |
| ***Patients with continuous sedative-administration stopped less than 24 h before or with EEG during continuous sedation***  n = 37 n = 23 | | | | |
| **Demographics and basic information:** |  |  |  |  |
| Female sex, n (%) | 11 (30) | 6 (26) | p=1.000^a^ | not included |
| Age [years], median (IQR) | 61 (53-70) | 60.0 (52-71) | p=0.867 | not significant |
| Out-of-hospital CPR, n (%) | 24 (65) | 16 (76) | p=0.556^a^ | not included |
| Initial rhythm: not shockable, n (%) | 10 (32) | 9 (50) | p=0.242^a^ | not significant |
| TROSC [min], median (IQR) | 12 (10-20) | 19 (13-22) | p=0.096^b^ | not significant |
| **NSE day 3** |  |  |  |  |
| [µg/l], median (IQR) | 21 (13-30) | 38 (24-110) | **p<0.001^b^** | not included |
| NSE > 90 µg/l, n (%) | 2 (5) | 7 (30) | **p=0.021^a^** | not significant |
| **Electrophysiology:** |  |  |  |  |
| **EEG** |  |  |  |  |
| Suppressed background without discharges, n (%) | 0 (0) | 5 (22) | **p=0.006^a^** | not included^†^ |
| GPDs on suppressed background, n (%) | 0 (0) | 3 (13) | p=0.052^a^ | not included^†^ |
| GPD on unsuppressed background, n (%) | 6 (16) | 6 (26) | p=0.508 | not significant |
| **SSEP** |  |  |  |  |
| SSEPs bilateral absent, n (%) | 0 | 4 (17) | **p=0.018^a^** | not included^†^ |

CPC – cerebral performance category at intensive care unit discharge; IQR interquartile range; tROSC – time to resuscitate spontaneous circulation; NSE – neuron specific enolase; CPR – cardio-pulmonal-resuscitation; ^a^Fisher´s exact (2-sided), ^b^Mann-Whitney-U test (2-sided); ^c^Pearson’s Chi-squared test (2-sided). Exp(B) – odds ratio; CI ­– confidence interval

**Clinical history of the patient with suppressed EEG background activity and favorable outcome after CPR**

The 67-year-old female patient was admitted to the intensive care unit after out-of-hospital resuscitation from cardiac arrest following pulmonal artery embolism. Time to return of spontaneous circulation was 20 minutes. The NSE at day 3 after CPR was 61 µg/l, but the patient had a neuroendocrine tumor of the sphenoidal sinus which might have elevated NSE values. SSEPs were without informative value.

The patient was sedated with midazolam and fentanyl for 3.5 days. The EEG recording analyzed in this study was performed 8 days after CPR, i.e. 4.5 days after cessation of continuous infusion of the sedative. No bolus of sedative medication was given or documented within 48 hours of the EEG recording. Plasma level of midazolam on the day of the EEG recording was not investigated. On the day of the EEG recording, the patient showed increased levels of urea (160 mg/dl; reference range 17 – 48 mg/dl) and vancomycin (40 mg/l; reference range through level 5 – 15 mg/l) due to acute kidney injury.

The patient was described as increasingly awake on the day prior to the EEG recording. As she was grabbing the tracheal tube, temporary physical restraint was necessary. On the morning of the day of the EEG recording the patient’s neurological status was documented as comatose. During the EEG recording, the patient showed unspecific withdrawal reactions to painful stimuli. Later that day, the patient again was moving spontaneously. The neurological status improved quickly in the days following the EEG examination. The next day, the patient was able to communicate non-verbally. Further follow-up EEGs were not part of the clinical routine. Thirty-seven days after CPR, the patient was discharged from ICU with a CPC of 1.
